# Detecting somatic mutations in genomic sequences by means of Kolmogorov–Arnold analysis

**DOI:** 10.1098/rsos.150143

**Published:** 2015-08-26

**Authors:** V. G. Gurzadyan, H. Yan, G. Vlahovic, A. Kashin, P. Killela, Z. Reitman, S. Sargsyan, G. Yegorian, G. Milledge, B. Vlahovic

**Affiliations:** 1 NSF Computational Center of Research Excellence and NASA University Research Center for Aerospace Device, NCCU, Durham, NC, USA; 2 Yerevan Physics Institute and Yerevan State University, Yerevan, Armenia; 3 Department of Pathology, Duke University Medical Center, The Preston Robert Tisch Brain Tumor Center at Duke, and Pediatric Brain Tumor Foundation Institute at Duke, Durham, NC, USA

**Keywords:** genome sequences, coding, randomness

## Abstract

The Kolmogorov–Arnold stochasticity parameter technique is applied for the first time to the study of cancer genome sequencing, to reveal mutations. Using data generated by next-generation sequencing technologies, we have analysed the exome sequences of brain tumour patients with matched tumour and normal blood. We show that mutations contained in sequencing data can be revealed using this technique, thus providing a new methodology for determining subsequences of given length containing mutations, i.e. its value differs from those of subsequences without mutations. A potential application for this technique involves simplifying the procedure of finding segments with mutations, speeding up genomic research and accelerating its implementation in clinical diagnostics. Moreover, the prediction of a mutation associated with a family of frequent mutations in numerous types of cancers based purely on the value of the Kolmogorov function indicates that this applied marker may recognize genomic sequences that are in extremely low abundance and can be used in revealing new types of mutations.

## 1. Introduction

To study mutations in the genomic sequences of cancerous tissues, we use the statistic introduced initially by Kolmogorov [1] and later developed by Arnold [2,3] in defining a degree of randomness (stochasticity) for a given sequence of real numbers. The universality of the method has been revealed in measuring the degree of randomness of finite sequences in theory of dynamical systems and in number theory [2]. This approach has been applied to physical problems, i.e. in the study of non-Gaussianities in cosmic microwave background radiation [4–6] and of X-ray galaxy clusters [7]. This method was instrumental in detecting the thermal trust effect (Yarkovsky–Rubincam effect) for the first time in the properties of satellites probing General Relativity [8].

## 2. Method

Let us briefly introduce the technique and the descriptors which were then applied to the genomic data. For {*X*_1_,*X*_2_,…,*X*_*n*_}*n* independent real-valued variables ordered in increasing manner *X*_1_≤*X*_2_≤⋯≤*X*_*n*_, the *cumulative distribution function* (CDF) is defined as *F*(*x*)=*P*{*X*≤*x*}[1–3].

The *empirical distribution function* *F*_*n*_(*x*) will be

2.1 $$F_{n}(x)=\left\{ \begin{array}{ll} 0, & x<X_{1};\\ \frac{k}{n}, & X_{k}\leq x<X_{k+1},\;k=1,2,\ldots ,n-1;\\ 1, & X_{n}\leq x. \end{array}\right.$$

Then the stochasticity parameter is defined as

2.2 $$\lambda_{n}=\sqrt{n}\underset{x}{\sup}|F_{n}(x)-F(x)|.$$

Kolmogorov’s theorem [1] states that for any continuous CDF *F* the following limit is converged uniformly:

2.3 $$\lim_{n\to \infty }P\{\lambda_{n}\leq \lambda \}=\Phi (\lambda ),$$

where the *Φ*(0)=0,

2.4 $$\Phi (\lambda )=\sum_{k=-\infty }^{+\infty }{\left(-1\right)}^{k}\;e^{-2k^{2}\lambda^{2}},\;\lambda >0,$$

and the distribution (Kolmogorov’s) *Φ* is independent of *F*. For small values of λ the following approximation yields:

2.5 $$\Phi (\lambda )\approx \frac{\sqrt{2\pi }}{\lambda }\;e^{-\pi^{2}/8\lambda^{2}}.$$

This method thus provides the measure of the degree of randomness (stochasticity) for sequences of *n* values within the interval of λ_*n*_ [0.3, 2.4] ([2,3], see also [9]).

## 3. Data

The following data have been used for the analysis. Gliomas are the most frequent malignant tumours of the central nervous system and are defined by WHO grade I to grade IV classification standards and histopathological features [10,11]. Application of novel techniques to elucidate the fundamental genetic mutations in grade II–III astroctyomas and grade IV glioblastomas is a critical next step in glioma research. Here, we interrogated exome data of 30 brain tumour patients from the Preston Robert Tisch Brain Tumor Center at Duke University as described previously [12]. The exomes of 30 patients were selected as they provided a large enough dataset to conduct our initial analysis. Each case contained four datasets corresponding to aligned paired end sequencing data files (both a forward and reverse file for each patient’s tumour and normal blood). Included in this study were 15 grade III astrocytomas and 15 grade IV glioblastomas. Samples on average yielded 36 and 32 somatic mutations for grade III astrocytoma and grade IV glioblastoma, respectively. Of particular interest to the general cancer community is the prevalence of highly recurrent mutations across all types of cancer. To this end, a list of highly recurrent mutations occurring in 23 genes commonly seen in cancer was used to interrogate the dataset (table 1). The selected genes are from a list of genes frequently mutated in cancer provided by Personal Genome Diagnostics (Baltimore, MD, USA). Next, we surveyed the exome data to identify any occurrence of these 407 highly recurrent mutations located within these 23 commonly mutated genes [13].

**Table 1.** Twenty-three genes commonly mutated in cancer.

gene symbol	gene description	transcript accession
ABL1	c-abl oncogene 1; non-receptor tyrosine kinase	X16416
AKT1	v-akt murine thymoma viral oncogene homologue 1	ENST00000349310
AKT2	v-akt murine thymoma viral oncogene homologue 2	NM 001626.3
ALK	anaplastic lymphoma receptor tyrosine kinase	NM 004304
BRAF	v-raf murine sarcoma viral oncogene homologue B1	NM 004333
CDK4	cyclin-dependent kinase 4	NM 000075.2
EGFR	epidermal growth factor receptor	NM 005228
ERBB2	v-erb-b2 erythroblastic leukaemia viral oncogene homologue 2	NM 004448
FGFR1	fibroblast growth factor receptor 1	NM 023110
FGFR3	fibroblast growth factor receptor 3	NM 000142
FLT3	fms-related tyrosine kinase 3	NM 004119
HRAS	v-Ha-ras Harvey rat sarcoma viral oncogene homologue	NM 005343
IDH1	isocitrate dehydrogenase 1 (NADP+); soluble	NM 005896.2
IDH2	isocitrate dehydrogenase 2 (NADP+); mitochondrial	NM 002168.2
JAK2	Janus kinase 2	ENST00000381652
KIT	v-kit Hardy-Zuckerman 4 feline sarcoma viral oncogene homologue	NM 000222
KRAS	v-Ki-ras2 Kirsten rat sarcoma viral oncogene homolog	NM 004985
MET	met proto-oncogene (hepatocyte growth factor receptor)	NM 000245
MET	met proto-oncogene (hepatocyte growth factor receptor)	NM 001127500
NRAS	neuroblastoma RAS viral (v-ras) oncogene homologue	NM 002524
PDGFRa	platelet-derived growth factor receptor; alpha polypeptide	NM 006206
PIK3CA	phosphoinositide-3-kinase; catalytic; alpha polypeptide	NM 006218.1
RET	ret proto-oncogene	NM 020975

## 4. Analysis

The data for 30 patients have been analysed in the following manner. First, for each dataset the cumulative distribution function was obtained for three combinations of nucleotides (guanine, adenine, thymine and cytosine (G,A,T,C), i.e. of codons. For comparison, in one- and two-based analyses of the same data, no CDF was possible to define due to large scatter in the frequency counts; however, it was possible for the three-base empirical distribution for each particular genomic sequence, followed by obtaining of the stochasticity parameter and then Kolmogorov’s function *Φ*. The fact that *Φ* was possible to define only for three-base CDF can be considered as a genuine feature linked to the nature of the genetic coding (first noted by cosmologist George Gamow in the 1950s) determined by the chemical properties of the molecules forming the nucleotides.

**Figure 1.**
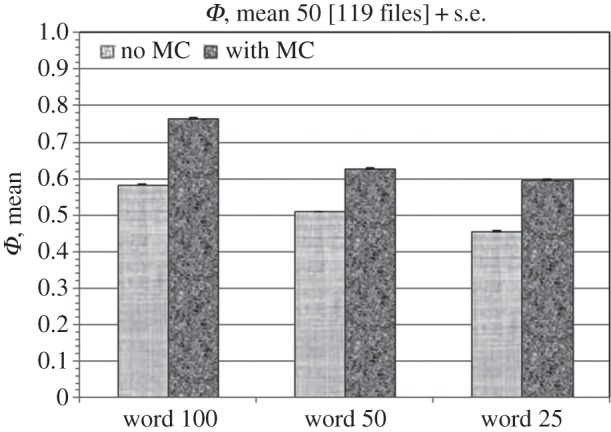
The function *Φ* averaged for rows with mutations (dark bars) and normal ones (light bars) averaged over 50 rows, for 100, 50 and 25-base rows (here denoted as word), correspondingly. Error bars correspond to standard errors.

The above-described exome data were represented in a format of over 50 million rows, of 100-base each, i.e. in total about 10^9^-valued sequences. We analysed such datasets for 30 patients; a block of four datasets, representing paired-end sequencing, was available for each patient, including those for blood and tumour, denoted as normal and with tumour, respectively. The following aim was inquired into: whether the Kolmogorov–Arnold (K–A) technique is able to distinguish the strings, i.e. sequence pieces of given length, with and without mutations, for a given sample of mutations. Figure 1 represents the results for computations for 100-base rows containing such mutations in all 119 blocks (dark column on the right; one file was corrupted), and the mean of the function *Φ* for 50 rows without mutations (light-coloured column on the left) in the same sequence where the former mutations have been located. The same procedure has been repeated for shorter, i.e. for 50 and 25-base strings with and without mutations (the right two double-columns, respectively). The shorter, 50-base strings were generated in the following manner: if the mutation is located completely either in the first or the second half of the 100-base string, then the corresponding halves were included in the analysis. In the case of partial location, the proper number of bases was included from either side, that is the mutations are included completely even at part passage to the next row. The case for 25-base strings was similar, while for no-mutation strings (rows) their initial 50 or 25-base parts were included in the analysis. For no-mutation rows their alignment, i.e. their position in the sequence, was not important. The error bars correspond to standard errors. The analysis was performed by means of a software created in *Pascal* in environment *Delphi* and intended to be made public in due course. The CPU time for one sequence (about 10^9^ nucleotides) is about 1 h for an i7, 2600 3.4 GHz processor of 6 GB memory.

**Figure 2.**
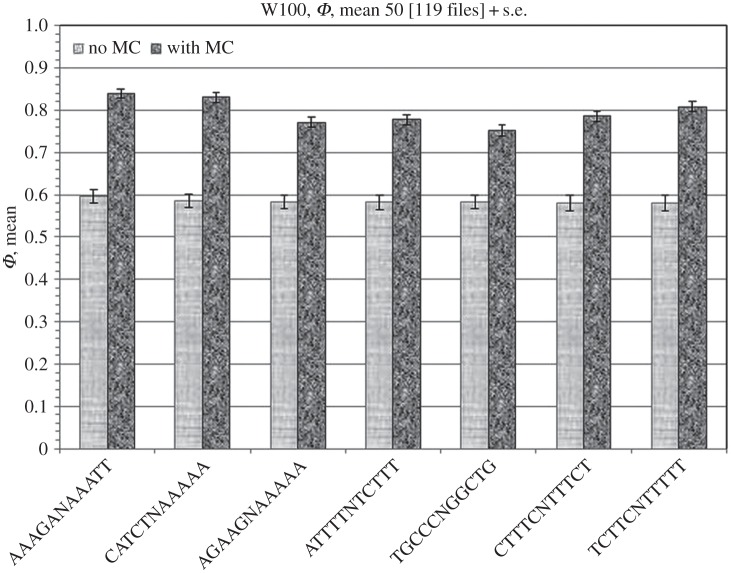
The same as in figure 1, but with the averaged values of the function *Φ* for 100-base rows for the highest recurrent specific mutations in the studied dataset as listed in table 2.

Then, rows containing the most frequent specific mutations in the same dataset of 30 patients (table 2) were analysed. Obviously, more frequent mutations provide higher statistics, and table 2 and figure 2 are represented to show the scales of the input frequency numbers versus the results. The mutations, i.e. the genes, the mutant positions and amino acid changes, are known for the codes listed in table 2, and from the individual mutation reports of the performed studies [12] one can list all mutations contained within each tumour; we intend to address these issues in further publications on the applications of the method discussed here. The results for the mean *Φ* with standard error bars are presented in figure 2. One can see that certain specific mutations can be distinguished by the value of mean *Φ*.

**Table 2.** The codes (MC) and frequency counts for seven highest recurrent mutations in the studied database.

N	MC	frequency
1	AAAGANAAATT	3032
2	CATCTNAAAAA	2569
3	AGAAGNAAAAA	2427
4	ATTTTNTCTTT	2409
5	TGCCCNGGCTG	2253
6	CTTTCNTTTCT	2200
7	TCTTCNTTTTT	2123

## 5. One step further: possibility for discovering new mutations

Up to now we were estimating the Kolmogorov function *Φ* for genomic sequence pieces (rows) with known mutations and without (those) mutations. We reveal the differences in the mean values of *Φ* for rows with and without those mutations. If so, then one can pose the inverse problem, namely, one can try to detect unknown mutations based on the value of *Φ*, if estimated blindly in a given genome sequence. In table 3, we show the results for a sample from the above studied database from genome with tumour (081T1): 11 rows (of over 50 million) with *Φ*>0.7 (only the rows with the number of unidentified nucleotides *n*≤3 have been taken into account) have been revealed with the codes given in table 3 as candidates for mutations. Obviously, certain parts of these cases can be just noise, i.e. without any association with real mutations; however, if at least part of this list when studied by conventional methods can be confirmed as associated with mutations, then one will have an explicit tool for detection of unknown mutations by means of this relatively simple (i.e. consuming little time and manpower) method. Certainly, the exhaustive answer to this question will need comprehensive parallel studies with different analysis methods. However, for now, for illustration, for the given sample of detected candidates for mutations, we performed the following. We sampled five reads from the aforementioned list of 11 rows in table 3 and aligned them to the human genome for further investigation. While none of these five reads matched to previously reported mutations in the COSMIC database, one of the reads aligned to a region of interest to oncogenomic laboratories. This read aligned to the transcriptional regulator ARID1A, an SWI/SNF family member that is frequently mutated in numerous types of cancers, including gastric, ovarian and pancreatic cancers [14–17]. Probability for a chance coincidence is less than 10^−5^ (assuming for simplicity an equipartition in the frequency counts, cf. table 2), thus proving the efficiency of the method for blind application to datasets using the previously calibrated *Φ*.

**Table 3.** The codes for candidates for mutations in a genome sequence with high *Φ* at given row numbers.

line N	MC
1186009	TTGTGNAAGGG
4073568	CCACGNCCTGG
11648505	ATACANAACGC
21240249	AAGGANACTGA
21827969	CAATTNGGGAA
33372865	GCCGGNCGCGG
34549019	TGGCCNAGAAG
36995074	TGAAGNGTTCT
42622891	TTGTTNTTTTA
43978737	AGAAANATATT
50647272	TGACTNAAAGG

Another potential application for this technique involves detection of rare variants of sequencing data, where the parameter *Φ* may recognize genomic sequences that are in extremely low abundance and warrant further investigation by the researchers.

## 6. Conclusion

The following basic conclusions can be drawn from the above analysis:

(i) the stability of the descriptor, that is small standard errors and hence high and stable confidence levels of the values of *Φ* for paired-end sequence rows both for normal, i.e. without mutations, and those with mutations;
(ii) the difference in values of *Φ* for rows with and without mutations;
(iii) the considered variations of string lengths still reflect the tendency; and
(iv) rows with certain mutations can be distinguished by means of the used marker.

The presented results demonstrate for the greater cancer research community the power of the K–A technique for identification of mutations in paired-end genome sequencing data. In addition to the significance of revealing the important nature of the difference in the degree of the randomness between the genome sequence with and without mutations, the method also has an important application potential. It may be applied to non-aligned sequencing segments, which may significantly simplify the procedure of finding segments with mutations and could speed up genomic research and its implementation in clinical diagnostics (cf. [18–20]).

Finally, the consideration of the inverse problem, namely, the revealing of a mutation associated with a tumour based purely on the computation of the value of the marker when blinded to the data, indicates that the latter may be used for detection of rare or new types of mutations.

## Data accessibility

The data files are located at http://www.impactjournals.com/oncotarget/index.php?journal=oncotarget&page=article&op=view&path[]=1505 and one can also get the data from http://www.ncbi.nlm.nih.gov/pubmed/24140581.
